# YOLO-SDL: a lightweight wheat grain detection technology based on an improved YOLOv8n model

**DOI:** 10.3389/fpls.2024.1495222

**Published:** 2024-11-19

**Authors:** Zhaomei Qiu, Fei Wang, Weili Wang, Tingting Li, Xin Jin, Shunhao Qing, Yi Shi

**Affiliations:** ^1^ College of Agricultural Equipment Engineering, Henan University of Science and Technology, Luoyang, Henan, China; ^2^ Science and Technology Innovation Center for Completed Set Equipment, Longmen Laboratory, Luoyang, Henan, China

**Keywords:** YOLOv8, wheat grain, lightweight model, object detection, computer vision

## Abstract

Wheat, being a crucial global food crop, holds immense significance for food safety and agricultural economic stability, as the quality and condition of its grains are critical factors. Traditional methods of wheat grain detection are inefficient, and the advancements in deep learning offer a novel solution for fast and accurate grain recognition. This study proposes an improved deep learning model based on YOLOv8n, referred to as YOLO-SDL, aiming to achieve efficient wheat grain detection. A high-quality wheat grain dataset was first constructed, including images of perfect, germinated, diseased, and damaged grains. Multiple data augmentation techniques were employed to enhance the dataset’s complexity and diversity. The YOLO-SDL model incorporates the ShuffleNetV2 architecture in its backbone and combines depthwise separable convolutions (DWConv) with the large separable kernel attention (LSKA) mechanism in its neck structure, significantly improving detection speed and accuracy while ensuring the model remains lightweight. The results indicate that YOLO-SDL achieves superior performance in wheat grain detection, balancing lightweight design and performance optimization. The model achieved a P of 0.942, R of 0.903, mAP50 of 0.965, and mAP50-95 of 0.859, with low computational complexity, making it suitable for resource-constrained environments. These findings demonstrate the efficiency of the ShuffleNetV2, DWConv, and LSKA structures. The proposed YOLO-SDL model provides a new technical solution for agricultural automation and serves as a reliable reference for detecting other crops.

## 1 Introduction

Wheat is one of the most widely cultivated food crops globally, and its grain yield and quality directly impact food security, making it a cornerstone of agricultural economies (Guo et al., 2023; Peng et al., 2022). During growth, harvesting, and storage, wheat grains can be affected by environmental factors or improper handling, leading to germination, damage, or disease, which compromise their quality (Ma et al., 2021). Thus, precise identification and detection of wheat grains, particularly distinguishing between different types of grains, is crucial for ensuring food safety and promoting sustainable agriculture (Que et al., 2023; Zhou et al., 2020). Moreover, grain detection plays a vital role in wheat breeding, enhancing breeding efficiency and aiding in the development of high-quality varieties (Misra et al., 2022).

Manual methods of detecting wheat grains suffer from inefficiency and subjectivity, failing to meet the demands of modern agriculture (Gao et al., 2022; Li et al., 2024c; Zhao et al., 2022). While traditional machine vision technology, which relies on computer-based image recognition and analysis, improves detection efficiency and accuracy, its generalization capability is limited. It tends to perform well on specific tasks but struggles to adapt to new ones, requiring expert intervention for feature extraction (Ji et al., 2022).

Recently, deep learning has made significant strides in object detection, particularly with deep neural networks excelling in image recognition and classification tasks (Feng et al., 2023; Han et al., 2023). Deep learning models for object detection are broadly categorized into two-stage and one-stage models. Two-stage models, such as the R-CNN series and Mask R-CNN, first generate candidate regions and then classify and localize these regions for accurate object detection (Fang et al., 2023; Li et al., 2024a). Li et al. (2022a) used a Faster R-CNN model to detect wheat spike in RGB images, achieving rapid and accurate detection with an average accuracy of 86.7%. Similarly, Ye et al. (2023) applied the Mask R-CNN model to visible-light images captured by uncrewed aerial vehicle to achieve rapid and accurate detection of individual cabbage plants. The study results demonstrated the superior performance of the Mask R-CNN model, with a detection accuracy of 99.5% and an overall average F1 score of 97.63%. Wu et al. (2020) combined transfer learning with the Faster R-CNN model for the detection and counting of wheat grains under complex environmental conditions. The results showed that the mean average precision (mAP) of the Faster R-CNN model was 0.91, and the model exhibited strong robustness. Although two-stage models exhibit high accuracy, they tend to be slower (Li et al., 2022b). In contrast, one-stage models like YOLO and SSD are widely favored for their speed and practicality, directly extracting image features to predict object categories and locations (Bacea and Oniga, 2023; Wan et al., 2024).

The YOLO series, a quintessential one-stage model, stands out for its exceptional performance and efficiency in object detection tasks (Wu et al., 2023; Zhang et al., 2024). However, while YOLO models are fast, there is still room to improve their detection accuracy. To optimize the YOLO algorithm, researchers have worked to enhance its accuracy and computational efficiency while reducing complexity and parameter count, aiming for a lightweight yet efficient model. For example, Ma et al. (2024) proposed an improved YOLOv8 model for wheat seed detection, incorporating shared convolutional layers and vision transformer with deformable attention mechanism, which resulted in a lightweight and efficient model with a mAP of 99.3%, a 16.8% improvement over YOLOv8. Similarly, Yao et al. (2024) improved the YOLOv7 model by integrating an efficient channel attention mechanism and a bi-directional feature pyramid network for detecting the germination rate of wild rice seeds, achieving detection accuracies of 94% and 98.2% in hydroponic and petri dish environments, respectively. Qing et al. (2024) proposed an enhanced YOLO-FastestV2 model for the detection of wheat spike counts. The model incorporated three attention mechanisms and a SimConv structure to enhance the model’s feature extraction capabilities and detection accuracy. The study demonstrated that the improved model achieved a precision (P) of 83.91%, recall (R) of 78.35%, average precision of 81.52%, and F1 score of 81.03%, indicating the overall best performance.

To enhance the accuracy and efficiency of wheat grain detection, this study proposes an improved YOLOv8n model, YOLO-SDL. The model adopts the ShuffleNetV2 architecture in its backbone, known for its computational efficiency, which not only boosts image processing speed but also significantly enhances detection accuracy. Additionally, depthwise separable convolutions (DWConv) replace the original convolutional modules in the neck of YOLOv8n, further reducing computational load, making the model more suitable for resource-constrained conditions. The large separable kernel attention (LSKA) mechanism is also integrated into the neck structure to improve the model’s ability to capture key object features, thereby enhancing detection precision. The contributions of this study are as follows: (1) constructing a high-quality dataset of wheat grains with various conditions, (2) conducting ablation experiments to validate the effectiveness of each improvement in the YOLO-SDL model, and (3) comparing the performance of YOLO-SDL with YOLOv5n, YOLOv6n, YOLOv8n, and YOLOv10n.

## 2 Materials and methods

### 2.1 Wheat grain dataset

**Figure 1:**
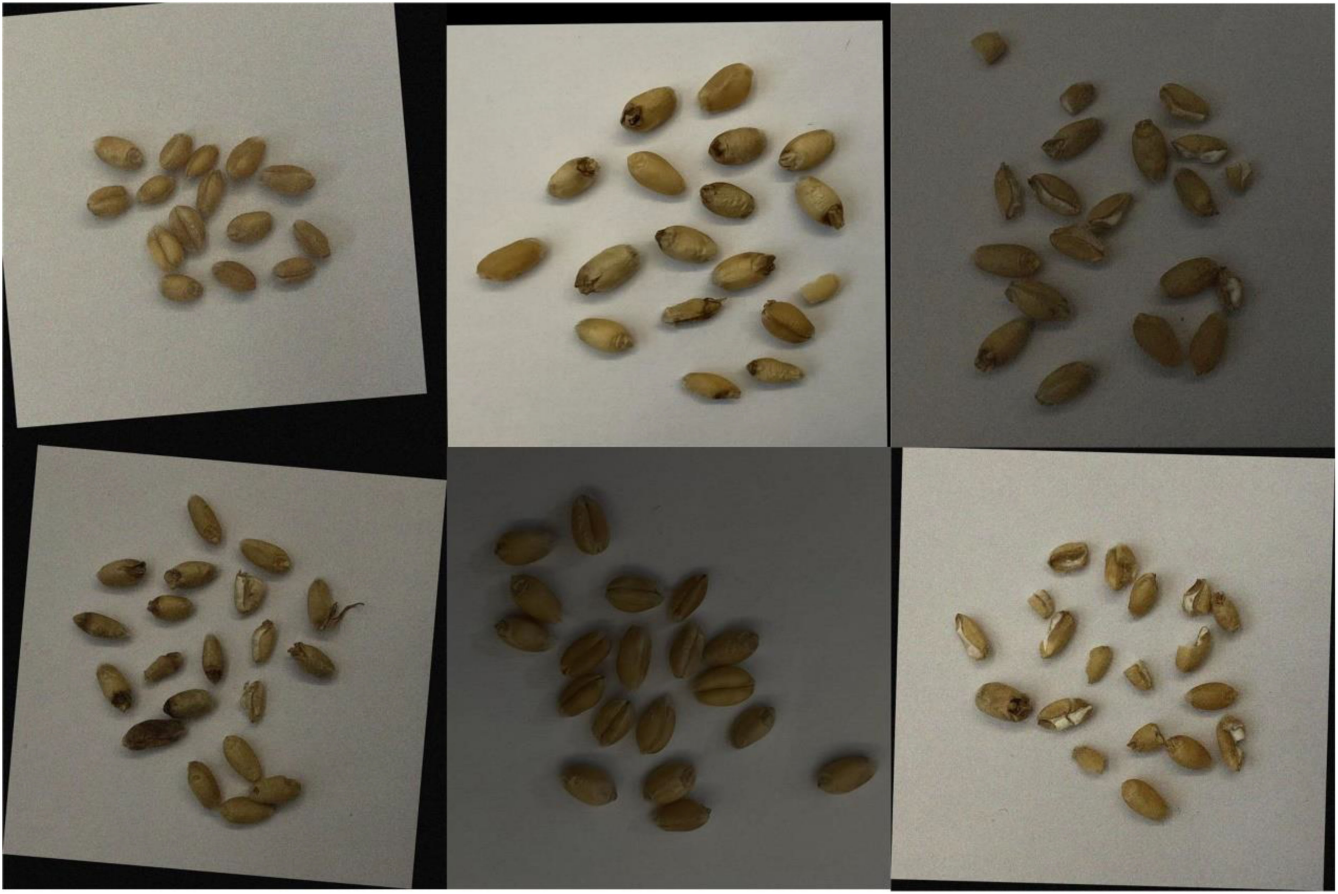
Sample images from the wheat grain dataset.

This study constructed a comprehensive and diverse wheat grain dataset, including images of perfect, germinated, diseased, and damaged grains, captured using high-resolution imaging equipment to ensure quality. After capturing the images, detailed bounding box annotations were made using the LabelImg software to ensure accurate identification of different grain conditions during model training. The dataset contains 9400 perfect grains, 7260 germinated grains, 835 diseased grains, and 1890 damaged grains, accounting for 48.49%, 37.45%, 4.31%, and 9.75% of the total, respectively. To further enhance the complexity and diversity of the dataset, various data augmentation techniques were employed, such as random scaling, rotation, flipping, noise addition, and adjustment of lighting intensity. These methods expanded the dataset’s size and richness, improving the model’s stability and generalization ability in recognizing wheat grains under different environments and conditions. The final dataset comprises 1170 images, divided into training, testing, and validation sets in an 8:1:1 ratio. Examples of wheat grain images are shown in **Figure 1**.

### 2.2 Improvement strategies

#### 2.2.1 YOLO-SDL model

The YOLOv8n algorithm builds upon the advantages of the YOLO series in terms of both accuracy and speed, with further optimizations to enhance performance. The YOLOv8n model introduces the C2f structure, which integrates feature maps from various depths, enabling the network to leverage both shallow and deep features simultaneously. This significantly enhances the model’s ability to recognize and capture object features (Jiang et al., 2024; Lu et al., 2024). The cross stage partial connections in the C2f structure optimize information flow through the network. By partially connecting different stages, the model not only improves feature representation but also strengthens its ability to detect objects in complex scenes. The neck of the YOLOv8n model employs the path aggregation network - feature pyramid network structure, which builds multi-scale feature fusion paths, allowing the model to handle objects of various scales more flexibly and efficiently. The detection head uses a decoupled structure that separates the classification and localization tasks, enabling independent optimization of both and improving detection accuracy and efficiency (Chen et al., 2024).

**Figure 2:**
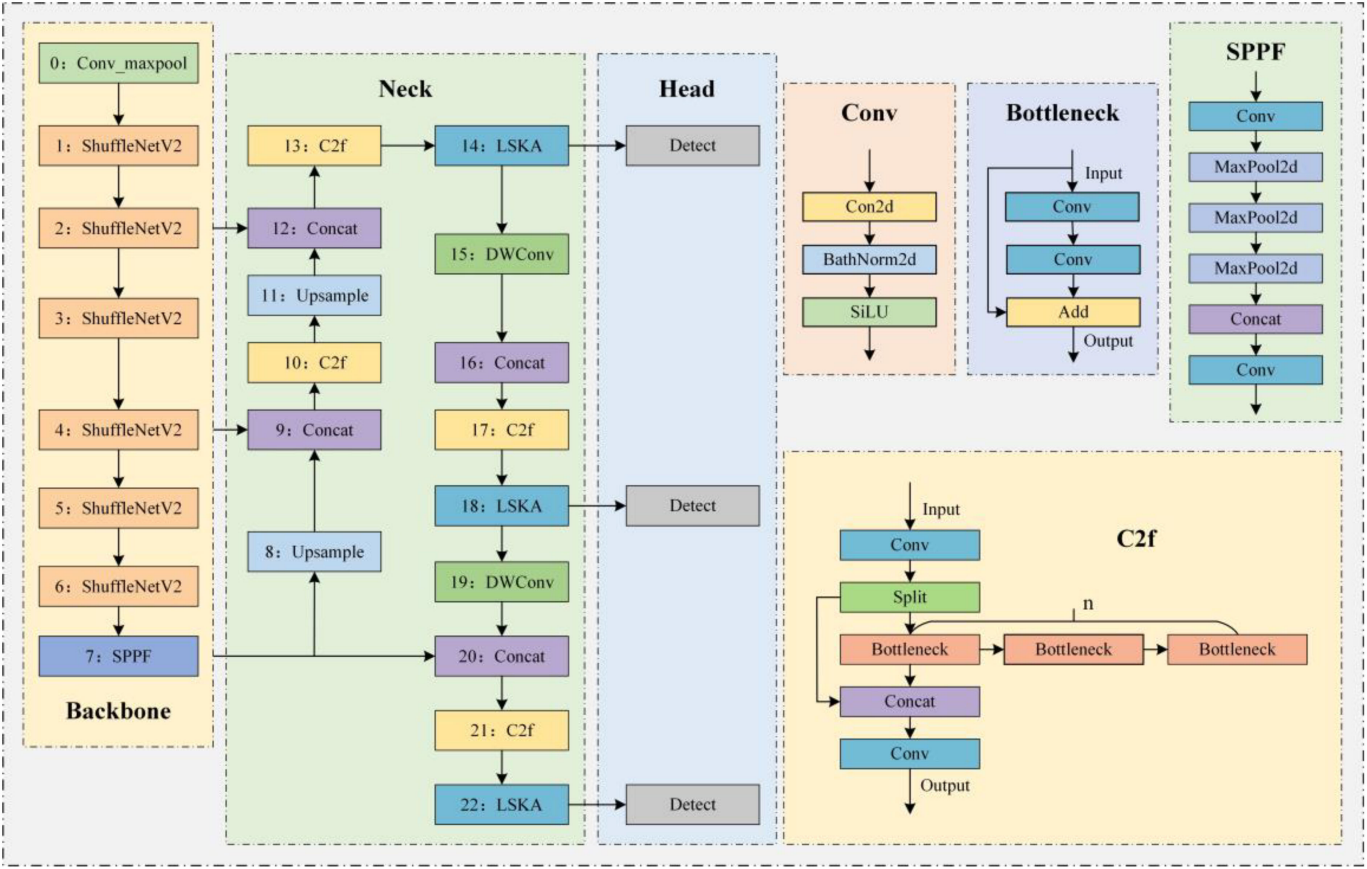
Structure of the YOLO-SDL model.

To further enhance detection accuracy while reducing computational complexity and parameter count, making the model more lightweight and efficient, this study proposes improvements to the YOLOv8n model. The structure of the improved model is shown in **Figure 2**. First, the ShuffleNetV2 architecture is introduced into the backbone of YOLOv8n, ensuring high precision while enabling fast object detection with lower computational cost. This is particularly crucial for tasks such as wheat grain detection, which often need to be conducted in environments like fields or warehouses where computational resources are limited. Additionally, we integrate DWConv and LSKA mechanisms into the neck of YOLOv8n. The DWConv structure significantly reduces the computational load, making the model more suitable for deployment on resource-constrained devices. The LSKA mechanism provides a more flexible feature extraction process, effectively enhancing the model’s detection accuracy. This aids the model in better capturing detailed characteristics of wheat grain under various environments to achieve accurate detection. By integrating improvements such as ShuffleNetV2 and DWConv, the model proposed in this study achieves structural lightweight and efficiency, and is functionally particularly suited for wheat grain detection tasks. It is capable of maintaining high detection accuracy while adapting to resource-constrained environments and meeting the demand for processing large volumes of image data.

#### 2.2.2 DWConv

**Figure 3:**
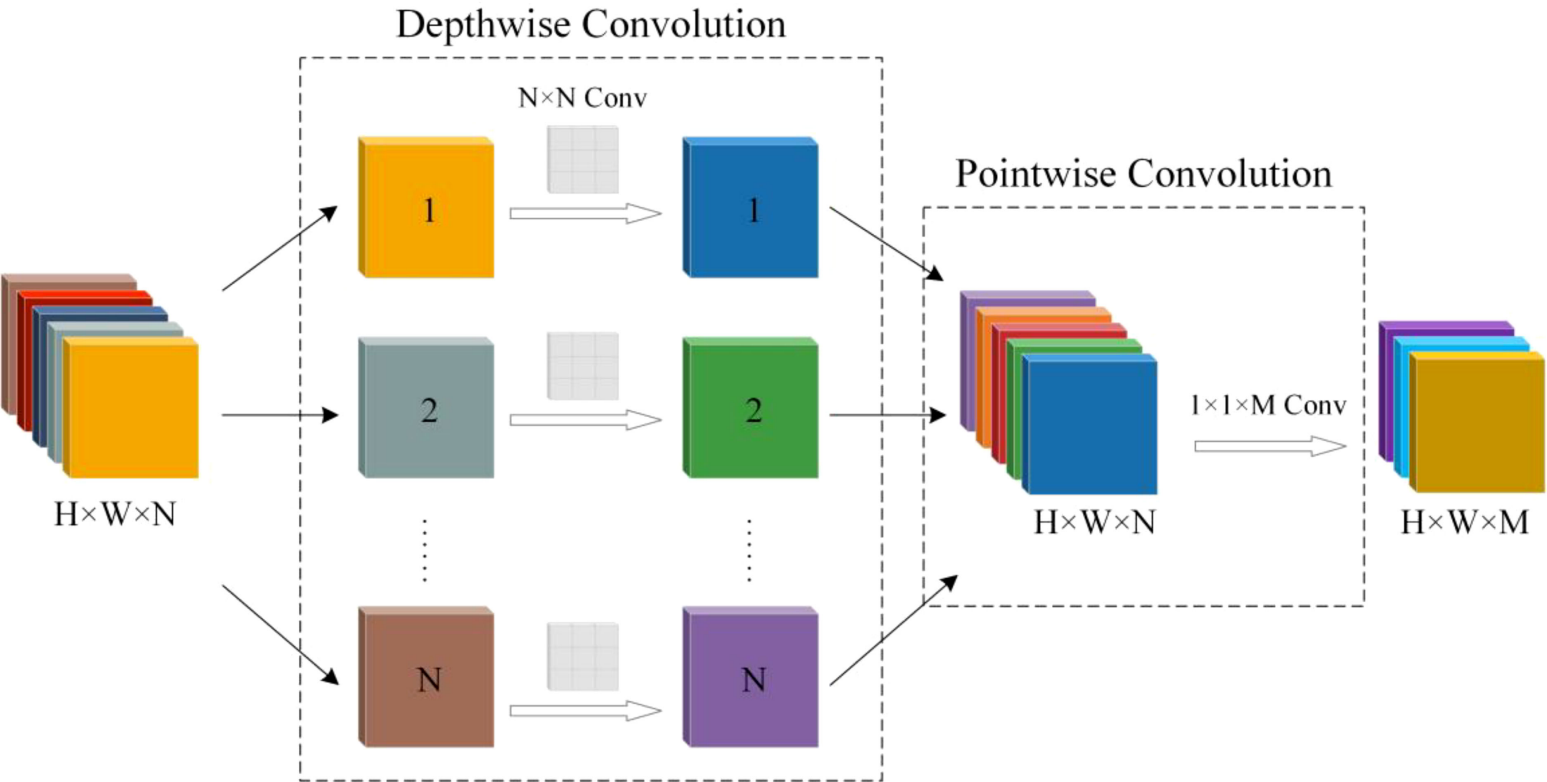
Structure of DWConv.

DWConv reduces the number of parameters in the model by decomposing a standard convolution into two processes: depthwise convolution and pointwise convolution, significantly lowering computational complexity (Balasundaram et al., 2023). During the depthwise convolution stage, each channel undergoes independent convolution operations, generating feature maps corresponding to the number of channels. This process enables the model to precisely recognize and capture the local features of each channel without a significant increase in the number of parameters. Pointwise convolution, which uses a 1×1 convolution kernel, integrates the feature maps produced by the depthwise convolution across channels, generating the final output feature map. The DWConv structure preserves the model’s ability to accurately capture spatial features while dramatically reducing its computational complexity and parameter count, resulting in a more lightweight model (Li et al., 2024b; Shi et al., 2024). DWConv replaces the original convolution structure in the YOLOv8n model, reducing model complexity and achieving further lightweight optimization. **Figure 3** illustrates the DWConv structure.

#### 2.2.3 ShuffleNetV2

**Figure 4:**
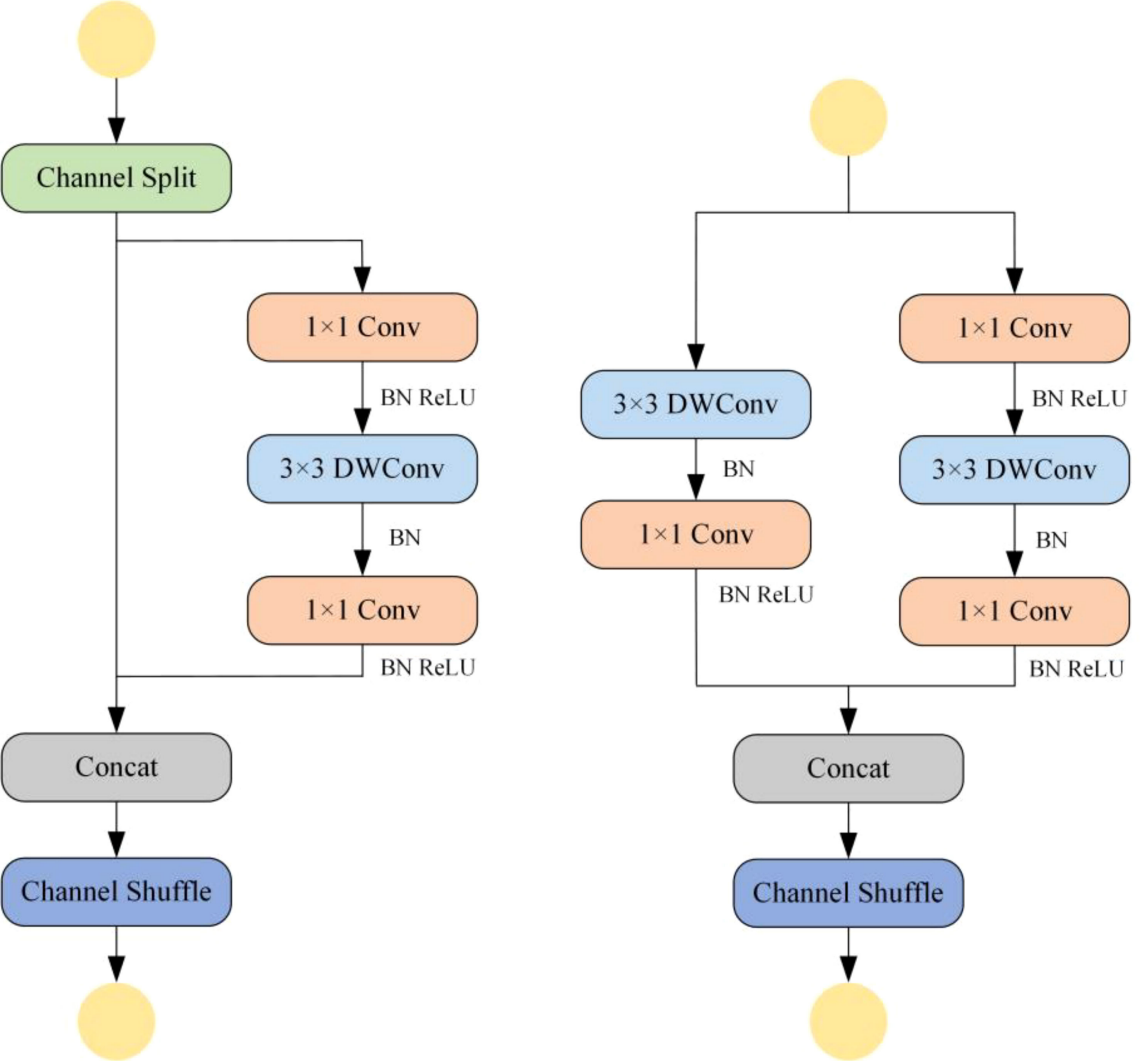
Structure of ShuffleNetV2.

ShuffleNetV2 is an efficient and lightweight convolutional neural network architecture that balances model complexity with accuracy (Thakuria and Erkinbaev, 2024). It employs group convolution to divide the input channels into groups, where independent convolution operations are performed within each group. This technique significantly reduces the number of parameters in the convolutional layers, thereby lowering the computational cost of the model. ShuffleNetV2 also incorporates an innovative channel shuffle mechanism, which enables effective feature fusion after the grouping process, enhancing the model’s ability to integrate features and improving detection accuracy. Moreover, the integration of DWConv further reduces the computational complexity of the model. Due to its lightweight and efficient nature, ShuffleNetV2 offers an effective solution for resource-constrained environments. ShuffleNetV2 exhibits a reduced computational parameter count relative to EfficientNet-B0, which is the smallest model within the EfficientNet series (Ding et al., 2023). It also demonstrates superior performance in both speed and accuracy compared to MobileNetV2, providing faster inference capabilities. These attributes render ShuffleNetV2 a highly suitable choice for applications within resource-limited environments (Ma et al., 2018). The channel shuffle mechanism and the design of the DWConv structure in ShuffleNetV2 provide better robustness when dealing with small objects, which is crucial for wheat grain identification and detection tasks. In this study, the ShuffleNetV2 architecture was applied to the backbone of the YOLO-SDL model, enabling it to adapt to environments with limited resources while maintaining fast and accurate object detection capabilities. **Figure 4** shows the structure of ShuffleNetV2.

#### 2.2.4 LSKA

**Figure 5:**
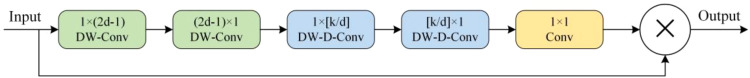
Structure of LSKA mechanisms.

LSKA is an innovative attention mechanism that decomposes a 2D convolutional kernel in the deep convolutional layers into two 1D convolutional kernels, performing horizontal and vertical convolution operations separately. This reduces the number of parameters and computational load while enhancing the model’s ability to capture image features (Lau et al., 2024). LSKA generates an attention map through a 1×1 convolution layer based on the 1D convolutions, which is used to adjust and enhance key information in the feature maps. The attention map is then multiplied pointwise with the original feature map to achieve adaptive optimization of the features (Zhong et al., 2024). Compared to traditional large kernel attention mechanisms, LSKA overcomes the computational and memory efficiency challenges associated with large convolutional kernels. It significantly reduces computational complexity, making it more efficient and flexible in object detection tasks, while providing more precise detection results. In this study, the LSKA mechanism was introduced into the neck network of the YOLOv8n model to further optimize its performance. **Figure 5** shows the structure of LSKA mechanisms.

### 2.3 Evaluation metrics

To assess the performance of different models in detecting wheat grains, this study employs P, R, mean average precision (mAP50), extended mean average precision (mAP50-95), and giga floating-point operations per second (GFLOPs) as evaluation metrics. P measures the proportion of correctly predicted positive samples out of all samples predicted as positive, as shown in Equation 1. R evaluates the proportion of actual positive samples that were correctly predicted by the model, as shown in Equation 2. mAP50 represents the mean average precision when the intersection over union (IoU) threshold is set at 50%, serving as a general indicator of the model’s overall performance, as shown in Equation 3. mAP50-95 is an extended version of mAP50 that considers a range of IoU thresholds (from 50% to 95% in increments of 5%), providing a more detailed performance evaluation across various levels of IoU, as shown in Equation 4. GFLOPs quantifies the capability of performing ten billion floating-point operations per second and serves as a pivotal metric for assessing the efficiency of deep learning models. It indicates the computational velocity of the models during both training and inference; a higher GFLOPs value signifies a model’s enhanced data processing capacity. As illustrated in Equation 5. The Frames per second (FPS) is an important indicator of the speed of model recognition and detection, which reflects the number of images processed by the model per second, the larger the FPS, the faster the processing speed of the model.


(1) $$P=\frac{TP}{TP+FP}$$

(2) $$R=\frac{TP}{TP+FN}$$

(3) $$mAP_{50}=\frac{1}{m}\sum_{i=1}^{m}AP_{i}$$

(4) $$mAP_{50-95}=\frac{1}{m}\sum_{i=1}^{m}\frac{1}{10}\sum_{j=1}^{10}AP_{i,j}$$

(5) $$GFLOPs=\frac{2*\left(K\times K\times C_{in}\times C_{out}\times H\times W+C_{out}\right)}{10^{9}}$$

(6) $$FPS=\frac{1}{Processing\;time\;per\;frame}$$

In this context, TP refers to the number of true positives, TN refers to the number of true negatives, FP refers to the number of false positives, and FN refers to the number of false negatives. *m* represents the number of classes in the dataset, *AP_i_* represents the average precision for class *i* when the IoU threshold is 0.5, and $AP_{i,j}$ represents the average precision for class *i* at IoU thresholds of 0.5+0.05×(j−1) . K×K denotes the size of the convolutional kernel, *C_in_* represents the number of input channels in the convolutional layer, *C_out_* represents the number of output channels in the convolutional layer, and H×W indicates the height and width of the feature map output by the convolutional layer.

## 3 Results

### 3.1 Experimental environment

The experiments were conducted on the Ubuntu 20.04 operating system, with Python 3.10 as the programming language, CUDA version 11.8, and PyTorch 2.0.1 as the deep learning framework. Jupyter was used as the IDE for developing the experiments. The CPU was an Intel(R) Xeon(R) Gold 5318Y @ 2.10GHz, and the GPU used was an NVIDIA A16 with 15GB of memory. During the network training process, this study sets the learning rate to 0.01 and the batch size to 200, with the loss function chosen as IoU. We employ a stochastic gradient descent optimizer with a momentum of 0.937, along with weight decay of 0.0005 to regularize the model and prevent overfitting. To more smoothly initiate the training process, we set up 3 warmup epochs, during which the initial momentum is set to 0.8 and the initial bias learning rate is set to 0.1. Such settings help to gradually enhance the model’s stability and convergence rate at the initial stage of training.

### 3.2 Ablation experiments

**Figure 6:**
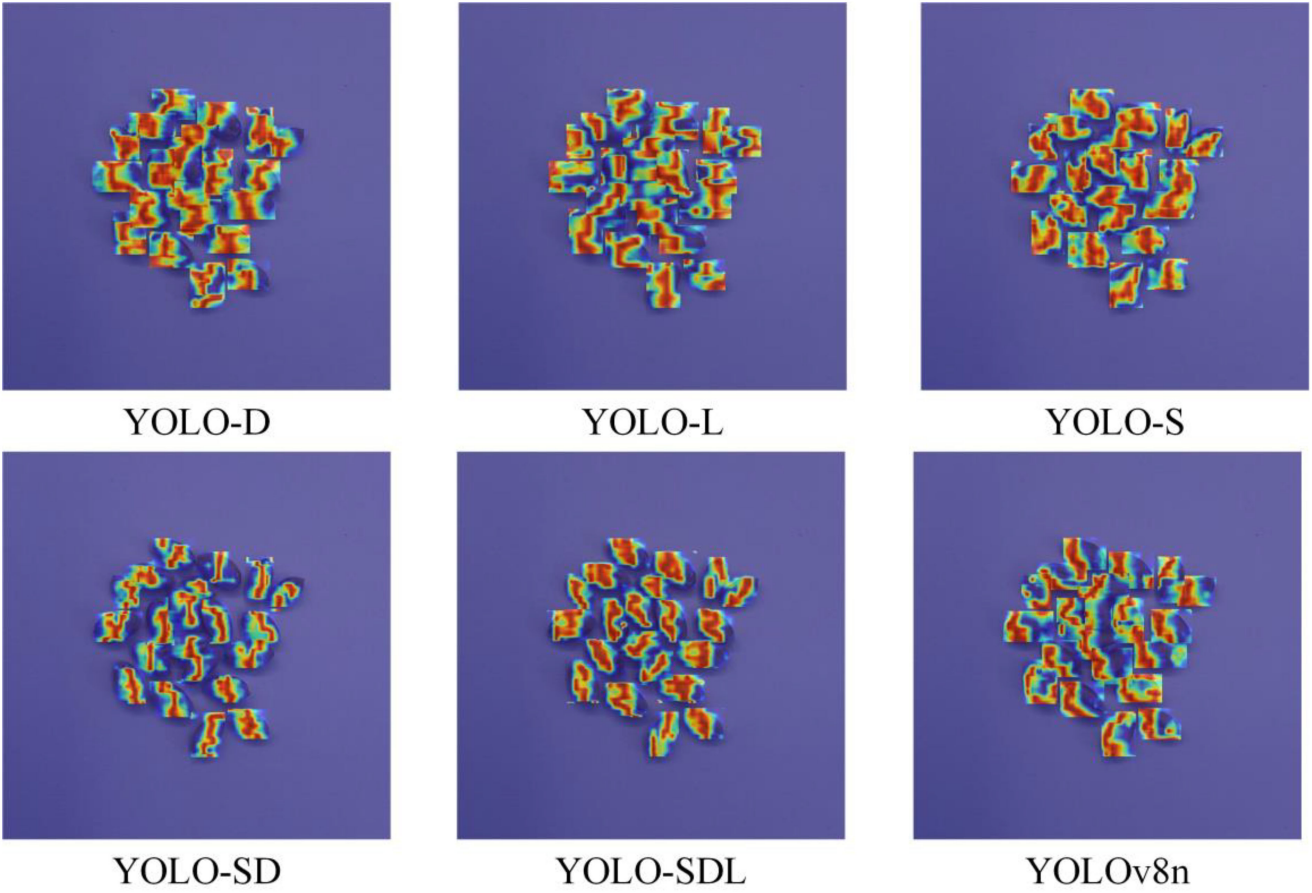
Heat map of ablation experiment results.

This study utilized the YOLOv8n model as a baseline and made several improvements by incorporating ShuffleNetV2, DWConv, and LSKA structures to enhance the accuracy of wheat grain detection. Ablation experiments were conducted to analyze the performance impact of each modification, with the results summarized in **Table 1**. In the ablation experiments, the results of different improved models on wheat grain feature recognition are shown in **Figure 6**.

**Table 1:** Accuracy results of ablation experiments.

Model	ShuffleNetV2	DWConv	LSKA	P	R	mAP50	mAP50-95
YOLOv8n	–	–	–	0.898	0.903	0.937	0.816
YOLO-S	√	–	–	0.909	0.917	0.954	0.834
YOLO-D	–	√	–	0.904	0.904	0.944	0.823
YOLO-L	–	–	√	0.904	0.926	0.952	0.832
YOLO-SD	√	√	–	0.911	0.917	0.960	0.848
YOLO-SDL	√	√	√	0.942	0.903	0.965	0.859

√ indicates that the module was used in this set of experiments; - indicates that the module was not used in this set of experiments.

The baseline YOLOv8n model achieved a detection performance of P = 0.898, R = 0.903, mAP50 = 0.937, and mAP50-95 = 0.816. The model had 3006623 parameters and a GFLOP count of 8.1. After introducing the ShuffleNetV2 architecture, the YOLO-S model improved its P to 0.909 and R to 0.917, with mAP50 and mAP50-95 reaching 0.954 and 0.834, respectively. The number of parameters and GFLOPs were reduced to 1835343 and 5.2, indicating that ShuffleNetV2 not only enhanced detection performance but also achieved significant model lightweighting. By incorporating the DWConv structure, the YOLO-D model achieved P = 0.904 and R = 0.904, with mAP50 and mAP50-95 improving to 0.944 and 0.823, respectively. The parameter count was 2824031, and GFLOPs were 7.9. While DWConv contributed to improved precision and target recognition ability, it offered slightly less performance improvement compared to ShuffleNetV2, suggesting potential for further optimization. With the introduction of the LSKA mechanism, the YOLO-L model saw increased complexity, with parameters growing to 3100255 and GFLOPs to 8.3. However, detection performance improved, with P = 0.904, R = 0.926, and mAP50 and mAP50-95 reaching 0.952 and 0.832, respectively, demonstrating the significant impact of LSKA on enhancing detection accuracy.

The YOLO-SD model, combining the strengths of ShuffleNetV2 and DWConv, achieved P = 0.911, R = 0.917, mAP50 = 0.960, and mAP50-95 = 0.848, with the parameter count reduced to 1652751 and GFLOPs to 4.9. These results indicate that the model achieved a high level of performance while further reducing its complexity. Finally, the YOLO-SDL model, which integrates ShuffleNetV2, DWConv, and LSKA, achieved the best overall performance with P = 0.942, mAP50 = 0.965, and mAP50-95 = 0.859. Although recall dropped slightly to 0.903, the parameter count was 1755215, and GFLOPs were 5.1. These results demonstrate the model’s ability to balance lightweight design with high accuracy and efficiency. According to the results demonstrated in **Figure 6**, it can be seen that the YOLO-SDL model focuses on the image region closer to the wheat grain, which has better recognition prediction effect.

This study significantly improved the accuracy of wheat grain detection by incorporating ShuffleNetV2, DWConv, and LSKA into the YOLOv8n model, achieving a balance between model lightweighting and computational efficiency. The results show that the ShuffleNetV2 architecture enhanced detection accuracy while significantly reducing model parameters and computational load. The DWConv structure provided modest accuracy improvements and reduced computational costs, though it still has room for further optimization. While the LSKA structure increased model complexity, it substantially improved detection accuracy. Among the various improved models, YOLO-SDL strikes the best balance between accuracy, parameter count, and computational efficiency, making it the optimal model for wheat grain detection.

### 3.3 Comparison of different models

**Figure 7:**
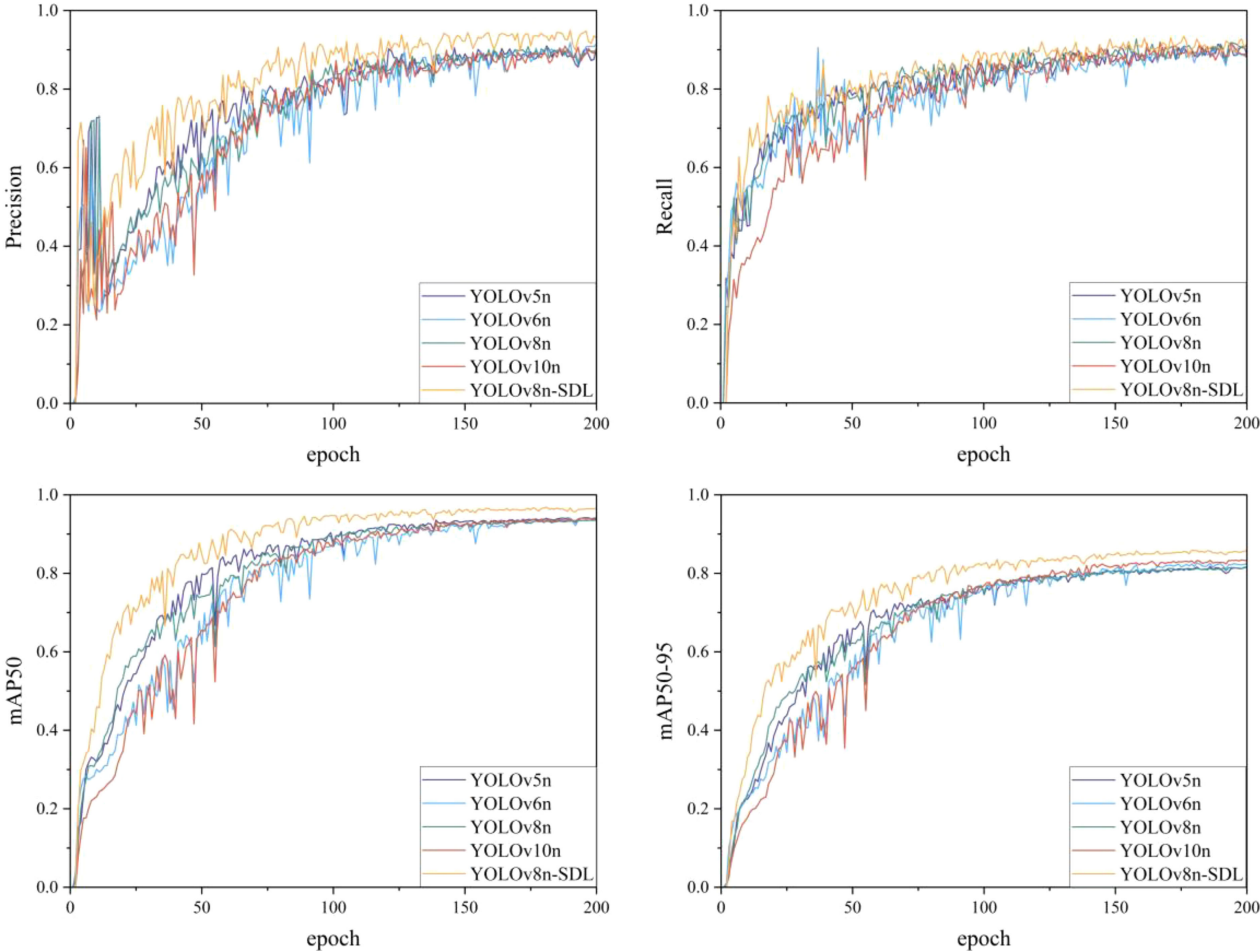
Comparison of detection accuracy variations across different models.

**Figure 8:**
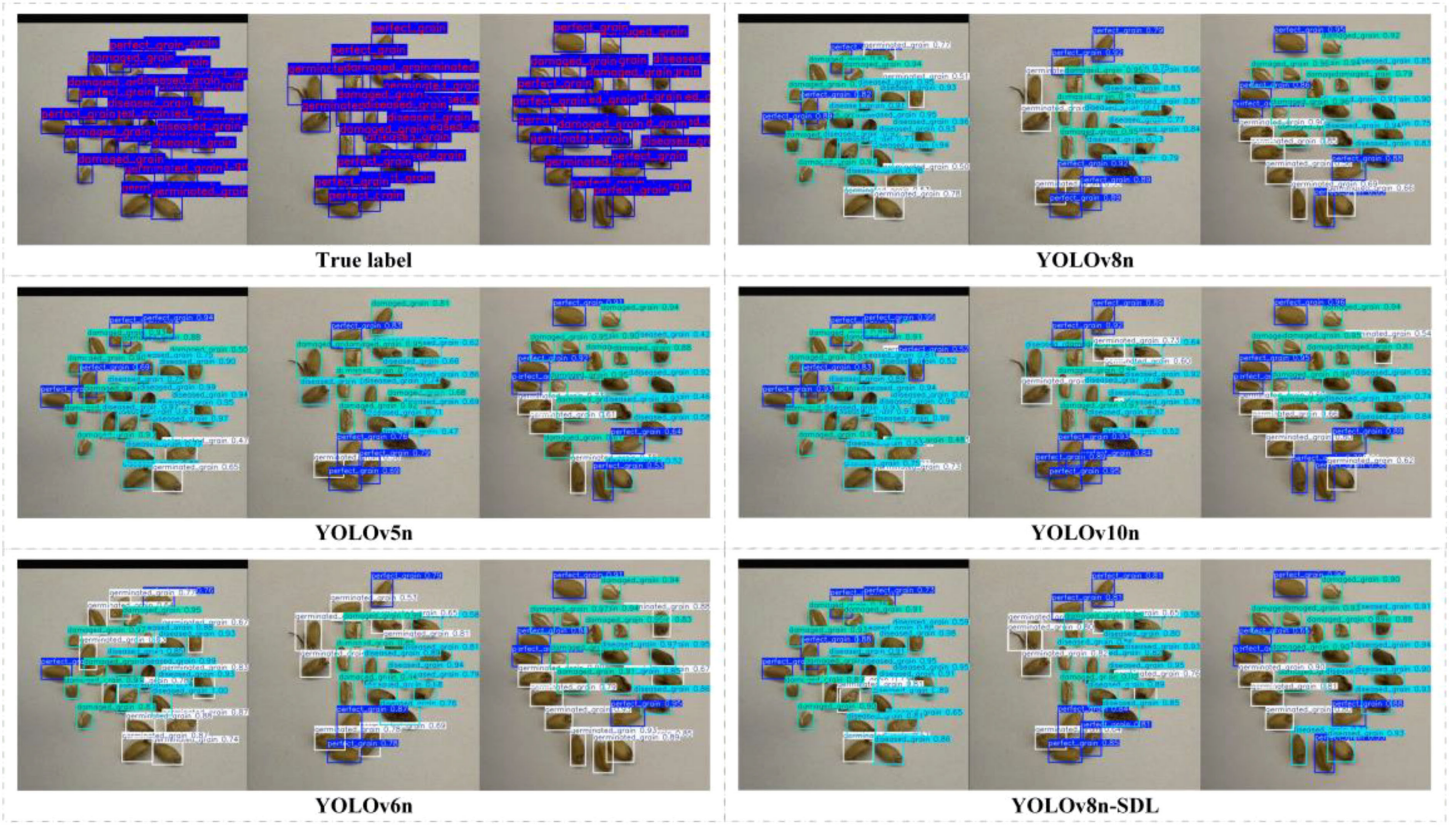
Comparison of detection results of different models.

This study compares the YOLO-SDL model with three other models from the YOLO series—YOLOv5n, YOLOv6n, and YOLOv10n—evaluating their accuracy and performance in wheat grain detection. The detection accuracy of the different models is summarized in **Table 2**, with the accuracy comparison trends shown in **Figure 7**, and the detection results comparison presented in **Figure 8**.

**Table 2:** Accuracy of detection results for different models.

Model	P	R	mAP50	mAP50-95	Parameters	GFLOPs	Speed (FPS)
YOLOv5n	0.900	0.887	0.940	0.817	2503919	7.1	156.250
YOLOv6n	0.902	0.888	0.938	0.827	4234239	11.8	106.383
YOLOv8n	0.898	0.903	0.937	0.816	3006623	8.1	140.845
YOLOv10n	0.895	0.887	0.940	0.834	2696366	8.2	175.439
YOLO-SDL	0.942	0.903	0.965	0.859	1755215	5.1	161.290

As shown in **Table 2**, the YOLOv5n model achieved relatively high P and R, with P = 0.900 and R = 0.887. Its mAP50 reached 0.940, while mAP50-95 was 0.817. The model also demonstrated a processing speed of 156.250 FPS, indicating a balance between high accuracy and fast processing. The YOLOv6n model had slightly higher P than YOLOv5n at 0.902, with R = 0.888, mAP50 = 0.938, and mAP50-95 improving to 0.827. It showed a real-time processing advantage with a FPS of 106.383. The YOLOv8n model had slightly lower P (P = 0.898), but R increased to 0.903, with mAP50 = 0.937 and mAP50-95 = 0.816. Its processing speed was 140.845 FPS, faster than YOLOv6n. The YOLOv10n model had the lowest P (P = 0.895) and R (R = 0.887), although its mAP50 and mAP50-95 were 0.940 and 0.834, respectively. It had the highest processing speed among all models at 175.439 FPS. In contrast, the YOLO-SDL model demonstrated a significant accuracy advantage in the wheat detection task, with P reaching 0.942, R at 0.903, and mAP50 and mAP50-95 of 0.965 and 0.859, respectively, making it the most accurate model among all tested. The YOLO-SDL also had a processing speed of 161.290 FPS, second only to YOLOv10n, indicating a fast image processing capability. From the perspective of computational complexity and efficiency, YOLO-SDL was the most lightweight model, with the smallest parameter count (1755215) and GFLOPs (5.1). In comparison, YOLOv6n had the highest computational cost, with 4234239 parameters and 11.8 GFLOPs. The YOLOv5n model was less complex, with 2503919 parameters and 7.1 GFLOPs, while the YOLOv8n and YOLOv10n models had intermediate levels of complexity.

The **Figure 6** shows the changes in accuracy for each model as the number of iterations increased. All models demonstrated an upward trend in accuracy over time, with the YOLO-SDL model maintaining the highest accuracy curve, consistently outperforming the other models. Additionally, as shown in **Figure 7**, the detection results indicate that the YOLO-SDL model was able to more accurately identify various types of wheat grains compared to the other models. In summary, the analysis demonstrates that appropriate adjustments and optimizations to the network structure can significantly enhance model performance. By integrating the strengths of ShuffleNetV2, DWConv, and LSKA, the YOLO-SDL model achieves the goal of reducing model complexity while maintaining high accuracy. This combination of lightweight design and high precision not only improves the model’s adaptability and practicality but also provides new insights for the fields of agricultural automation and smart agriculture.

## 4 Discussion

This study introduces improvements to the YOLOv8n model by incorporating ShuffleNetV2, DWConv, and LSKA structures, with the goal of optimizing detection accuracy, reducing the model’s parameter count, and lowering computational complexity. These enhancements aim to improve the model’s performance in wheat grain detection tasks while achieving a balance between model lightweighting and computational efficiency. Through ablation experiments and comparative analysis with different models, this research demonstrates that the YOLO-SDL model is the optimal solution for wheat grain detection, offering high-precision results while maintaining a lightweight design.

The YOLO-SDL model leverages the ShuffleNetV2 architecture as its backbone, significantly improving detection accuracy while greatly reducing both the model’s parameter count and computational load. ShuffleNetV2, an efficient lightweight network architecture, uses grouped convolution, channel shuffle mechanisms, and DWConv to capture rich feature information while minimizing computational complexity. These lightweight features are especially valuable in resource-constrained environments. Shang et al. (2023) demonstrated in their study on apple flower detection that ShuffleNetv2 possesses significant advantages in terms of lightweight design, accuracy, and adaptability, making it an ideal choice for object detection in resource-constrained environments. Additionally, the introduction of DWConv and LSKA structures in the neck of the YOLO-SDL model further enhances its performance. While the DWConv structure does not provide as significant a precision boost as ShuffleNetV2, it plays a crucial role in reducing computational costs. By lowering the number of parameters and computational load in convolution operations, DWConv improves both efficiency and accuracy. Guo et al. (2024) showed that replacing the convolutional modules in the YOLOv8s network with DWConv modules can reduce the complexity of the network, proving to be an effective lightweight strategy. Although LSKA increases model complexity, it also delivers significant accuracy improvements. By decomposing large kernels into smaller, more manageable parts, LSKA can effectively model global information, which is crucial for distinguishing between wheat grains and other components such as husks and stalks. The LSKA mechanism enhances the model’s ability to recognize features in wheat grains by adaptively optimizing local spatial attention within the feature maps. Gao et al. (2024) pointed out in their research aimed at improving the efficiency and accuracy of young citrus fruit detection that the LSKA mechanism enhances the model’s ability to fuse features across different scales, thereby improving detection accuracy. The inclusion of DWConv and LSKA in YOLO-SDL boosts detection accuracy, thereby improving overall model performance.

In the model comparison analysis, YOLO-SDL demonstrated clear performance advantages in wheat grain detection tasks. The model achieved the highest values for P, R, mAP50, and mAP50-95 compared to other YOLO series models. From a computational complexity and efficiency standpoint, YOLO-SDL is the most lightweight model, outperforming the others in terms of parameter count and GFLOPs. These findings suggest that appropriate network adjustments and optimizations can significantly enhance overall detection performance while maintaining a lightweight design.

In conclusion, the YOLO-SDL model introduced in this study has demonstrated superior performance in wheat grain detection tasks. By combining the strengths of ShuffleNetV2, DWConv, and LSKA, the model achieves significant improvements in accuracy while balancing lightweight design and performance optimization. These results offer new approaches and methods for agricultural automation and smart farming. Future research could explore further reductions in model complexity, optimize the network structure, and enhance the model’s adaptability in resource-limited environments, advancing the development and application of agricultural automation technologies.

## 5 Conclusion

This study developed an improved YOLOv8n model, termed YOLO-SDL, for detecting various types of wheat grains. Through in-depth analysis and enhancement of the YOLOv8n model, a lightweight, efficient, and highly accurate wheat grain detection model was constructed. By introducing ShuffleNetV2, DWConv, and LSKA structures, the YOLO-SDL model significantly improves the capture of features across different wheat grain types while maintaining high precision, lightweight design, and computational efficiency. The results show that the YOLO-SDL model excels in detection accuracy, achieving a P of 0.942, R of 0.903, mAP50 of 0.965, and mAP50-95 of 0.859, outperforming YOLOv8n and other YOLO series models in both lightweight design and computational efficiency. The YOLO-SDL model’s advantages in parameter count and computational complexity make it highly suitable for deployment in resource-constrained environments, which is particularly relevant for real-world agricultural automation and intelligent systems. The outstanding performance of the YOLO-SDL model is attributed to the introduction of ShuffleNetV2, DWConv, and LSKA structures. The ShuffleNetV2 architecture, through its use of grouped convolution and channel shuffle mechanisms, maintains high accuracy while reducing computational costs. The DWConv structure, by decomposing standard convolutions into depthwise and pointwise convolutions, further reduces parameter count and computational complexity. Finally, the LSKA mechanism, by decomposing 2D convolutional kernels into 1D kernels, achieves adaptive feature optimization, enhancing the model’s ability to capture image features and improving detection accuracy.

The YOLO-SDL model demonstrates excellent performance in wheat grain detection tasks, improving both accuracy and efficiency while achieving a lightweight design. This provides a novel technical solution for agricultural automation and intelligent farming. Future research will continue to explore and optimize the model’s structure to extend its applicability to a wider range of agricultural scenarios, driving further advancements in agricultural technology.

## Data Availability

The raw data supporting the conclusions of this article will be made available by the authors, without undue reservation.
